# Glucosamine delays the progression of osteoporosis in senile mice by promoting osteoblast autophagy

**DOI:** 10.1186/s12986-022-00688-y

**Published:** 2022-11-08

**Authors:** Wei Su, Chen Lv, Lingtuo Huang, XiaoHang Zheng, Shengwu Yang

**Affiliations:** Department of Orthopedics, The First Affiliated Hospital of Wenzhou Medical University, Wenzhou, 325000 Zhejiang China

**Keywords:** Glucosamine, d-Galactose, Senile osteoporosis, Osteogenesis, Autophagy

## Abstract

**Background:**

Senile osteoporosis (SOP) is one of the most prevalent diseases that afflict the elderly population, which characterized by decreased osteogenic ability. Glucosamine (GlcN) is an over-the-counter dietary supplement. Our previous study reported that GlcN promotes osteoblast proliferation by activating autophagy in vitro. The purpose of this study is to determine the effects and mechanisms of GlcN on senile osteoporosis and osteogenic differentiation in vivo.

**Methods:**

Aging was induced by subcutaneous injection of d-Galactose (d-Gal), and treated with GlcN or vehicle. The anti-senile-osteoporosis effect of GlcN was explored by examining changes in micro-CT, serum indicators, body weight, protein and gene expression of aging and apoptosis. Additionally, the effects of GlcN on protein and gene expression of osteogenesis and autophagy were observed by inhibiting autophagy with 3-methyladenine (3-MA).

**Results:**

GlcN significantly improved bone mineral density (BMD) and bone micro-architecture, decreased skeletal senescence and apoptosis and increased osteogenesis in d-Gal induced osteoporotic mice. While all effect was reversed with 3-MA.

**Conclusion:**

GlcN effectively delayed the progression of osteoporosis in senile osteoporotic mice by promoting osteoblast autophagy. This study suggested that GlcN may be a prospective candidate drug for the treatment of SOP.

## Introduction

Osteoporosis is a common orthopedic disease in elderly population [1]. According to the available epidemiological data, there are about one-third of the world's elderly persons are suffering from osteoporosis, which makes them vulnerable to brittle fractures and reduces quality of life [2]. Senile osteoporosis (SOP) is characterized by loss of bone mass and destruction of bone microstructure, which is caused by an imbalance between bone formation and bone resorption and mainly due to the reduction of osteogenic ability [3, 4]. But the mechanisms is not clear, may be associated with malnutrition [5], low sex hormones [6], high endogenous glucocorticoids [7] and accumulation of harmful substances [8].

Glucosamine (GlcN) is an amino monosaccharide, which is an important component of the cartilage matrix [9], used by consumers as an over-the-counter dietary supplement to anti-osteoarthritis and improve the joint function [10]. Moreover, there are a few reports about GlcN promoting osteogenesis. Jiang et al. found that GlcN protects osteoblasts from oxidative damage in vitro [11]. Ali et al. found that ovariectomized rats helps to preserve bone mass and biomechanical properties by feeding GlcN [12]. Kalbe et al. reported that GlcN accelerates the early repair of tibial fractures in rats by increasing the activity of osteoblasts and promoting new bone formation [13]. Therefore, it is interesting to explore whether GlcN has a therapeutic effect on senile osteoporosis which caused by reduced osteogenesis.

Our previous study reported that GlcN promotes the proliferation of osteoblasts in vitro by up-regulating autophagy [14]. However, the effect of GlcN on senile osteoporosis is not clear, nor is it known whether autophagy is involved. Autophagy, as an internal equilibrium mechanism to maintain eukaryotic homeostasis, plays an important role in many diseases and physiological processes [15]. Lenoir et al. revealed the protective role of autophagy in models of senescence and acute kidney injury [16]. Habieb et al. suggested that inhibition of autophagy accelerates mouse hepatic cells senescence induced by d-Galactose or γ-Irradiation [17]. Therefore, autophagy helps cells adapt to various external stimuli and maintain cell homeostasis. The reduction of autophagy accelerates cell senescence, while the promotion of autophagy plays an anti-senescence effect [18, 19]. At present, the role of autophagy in the occurrence and development of metabolic diseases has attracted more and more attention [20]. In addition, autophagy plays an important role in age-related bone loss, which helps prevent fragility fracture [21]. Recently in vivo research showed that knocking out the Atg7 gene, one of the most important genes associated with autophagy, inhibits autophagy in 6-month-old mice, leading to a decline in osteoblast counts and age-inconsistent osteoporosis [22]. Another study reported that mouse osteoblast autophagy is involved in the process of bone mineralization, and inhibition of osteoblast autophagy simulates senescence in mice [23].

Based on these findings, we hypothesize that GlcN reduces bone loss in elderly mice and delays the progression of senile osteoporosis by promoting osteoblast autophagy. We believe that this study will provide a solid foundation for the clinical application of GlcN in the treatment of senile osteoporosis.

## Methods and material

### Main reagents

d-Gal and GlcN were procured from Sigma-Aldrich (Darmstadt, Germany). 3-Methyladenine (3-MA) was purchased from Selleckchem (Houston, TX, USA). ELISA kits for cytokines IL-1β and IL-6 were procured from Boster (Wuhan, Hubei, China). Malonaldehyde (MDA) and superoxide dismutase (SOD) assay kit was purchased from Jiancheng Bioengineering (Nanjing, Jiangsu, China).The cDNA was synthesized by reverse transcription using a cDNA synthesis kit (Takara, Japan), and was analyzed with SYBR Green Real-time PCR kit (Novizan Biotechnology, Nanjing, Jiangsu, China).The antibodies included LC3, Beclin-1, P62, RUNX2, OCN, BMP2, Bcl-2, BAX and Cleaved Caspase-3 were purchased from Cell Signaling Technology (Danvers, MA, USA), and the antibodies to Ki-67 and p16 were all obtained from Beyotime (Shanghai, China). The antibody to β-actin and the second antibody conjugated HRP (goat-anti-mouse and goat-anti-rabbit) were purchased from Proteintech (Chicago, IL, USA). The Alkaline phosphatase (ALP) staining kit, Goldner trichrome staining kit and Tartrate-resistant acid phosphatase (TRAP) staining kit were purchased from Solarbio (Beijing, China).

### Experiment animals and groups

Specific pathogen-free (SPF) grade male C57BL/6 mice (6–8 weeks of age) were used for in vivo experiment. All animal experiments were approved by the Animal Experimental Ethics Committee of Wenzhou Medical University, and complied with Chinese laws and regulations (wydw2019-0537). These mice were bred and maintained at the laboratory animal center of Wenzhou Medical University. Mice were co-housed in a controlled environment with free access to food and water. After 1 week of adaptive feeding, the mice were randomly and equally divided into different groups (n = 8). The experimental senile osteoporosis model was induced by the subcutaneously administered of d-Gal (150 mg/kg/day) fresh dissolved in PBS for 12 weeks. 12 weeks after the modeling, the mice were treated according to their respective grouping. d-Gal group were continued to treat with d-Gal (150 mg/kg/day). GlcN group were additionally treated with GlcN (9 mg/Kg/day). 3-MA group were additionally treated with 3-MA (4 mg/kg/day). GlcN + 3-MA group were additionally treated with GlcN (9 mg/Kg/day) and 3-MA (4 mg/kg/day). All drugs were dissolved in PBS. Equal volume solvent blank (PBS) was used as control. After the experiment, all mice were euthanized to collect specimens in each group.

### Serum biochemical test

Whole blood was gathered in each group (n = 8) by cardiac puncture into syringes. Serum samples were separated after centrifugation at 3000 rpm for 15 min under 4 °C. Serum IL-1β and IL-6 were selected as indicators of senescence in mice, and the levels of serum IL-1β and IL-6 were measured using a commercial ELISA kit to reflect the senescence of mice. Serum MDA and SOD were selected as indicators of oxidative stress in mice, and the levels of serum MDA and SOD were determined using a MDA and SOD test kit to determine the degree of oxidative stress in vivo. All of the procedures were performed, according to the manufacturer’s protocols.

### Micro-CT analysis

The bone trabecula microstructures of the distal right femur of mice in each group (n = 8) were determined by micro-CT (skyscan1176, Bruker, Germany). The scanning conditions were consistent (resolution: 18 μm; source voltage: 65 kV; source current: 385 μA). The bone trabeculae of each scanning sample (mouse femur) was selected starting form 1.5 mm below the growth plate and 2 mm long, as well as the bone cortex starting from 5 mm below the growth plate and 2 mm long. A series of 2D image data were gathered and appropriate gray values were selected for 3D reconstruction of bone trabecula and bone cortex. Each micro-CT section delineated the trabecular area of bone. The parameters of bone trabecula included bone mineral density (BMD), bone volume/total volume (BV/TV), number of trabecular (Tb.N), trabecular thickness (Tb.Th), trabecular spacing (Tb.Sp) and structural model index (SMI) were recorded with the built-in software.

### Three-point bending test

The right femur strengths of mice (n = 8 each group) were detected by three-point bending test using an MTS 858 Mini Bionix System (Wenzhou institute of Biomaterials and Engineering, Wenzhou, Zhejiang, China). The femur was placed in a holder to ensure that the applied force was perpendicular to the midpoint of the femur diaphysis. The load distance was 8 mm and the load rate was 3 mm/min until femoral fracture, then drawing the stress–strain curves. The parameters of femur, including bone stiffness, maximum load, elastic load and fracture load, were calculated according to the corresponding formulas.

### Western blot analysis (WB)

The lower left limbs of the mice (n = 8 each group) were isolated, and the excess soft tissue were stripped. The bone tissues with a pre-cooling at − 80 °C overnight were ground in a precooled mortar under liquid nitrogen flow, and homogenized in RIPA buffer supplemented with protease inhibitor. Equal amounts of protein from each group were separated by SDS-PAGEs and transferred to PVDF membranes. The membranes were blocked with 5% milk in TBS-T for 2 h at room temperature, briefly washed with TBS-T, incubated with primary indicated antibodies overnight at 4℃. After washed three times with TBS-T, the membranes were incubated with secondary antibodies for 2 h at room temperature. The signal was finally visualized using the enhanced chemiluminescence (ECL) detection system. The antibody β-actin was used as an internal control.

### Real-time quantitative polymerase chain reaction (qRT-PCR)

Tibia specimens were used for qRT-PCR. Treated tissues (n = 8 each group) were milled in liquid nitrogen and then used for RNA extraction. Total RNA was isolated using Trizol reagent (Invitrogen, Carlsbad, CA, USA). SYBR Green Real-time PCR was performed using an ABI 7500 Real-time PCR systems (Thermo, Foster City, CA, USA). β-actin was used as an internal standard. The specific forward and reverse primers sequences were shown in Table 1.

**Table 1:** Primer sequence

Gene	Sequence
Beclin-1	F. 5′AGCACGCCATGTATAGCAAAGA3′
	R. 5′GGAAGAGGGAAAGGACAGCAT3′
p62	F. 5′CGTTTGACGGAAGGTAAAT3′
	R. 5′TCATCAGCGGGCTGTATC3′
Bcl-2	F. 5′TGTTGTTCAAAGGGA TTCA3′
	R. 5′GGCTGGGCACATTTACTGTT3′
Bax	F. 5′GGGGACGAACTGGACAGTAA3′
	R. 5′CAGTTGAAGTTGCCGTCAGA3′
Cleaved caspase-3	F. 5′CAAACTTTTTCAGAGGGGATCG3′
	R. 5′GCATACTGTTTCAGCATGGCAC3′
BUNX2	F. 5′CCTGAACTCTGCACCAAGTCCT3′
	R. 5′GTGACATTGTCCATCATTGGGTA3′
OCN	F. 5′TGAGAGCCCTCACACTCCTC3′
	R. 5′ACCTTTGCTGGACTCTGCAC3′
BMP-2	F. 5′ACTACCAGAAACGTGGGAA3′
	R. 5′GCATCTGTTCTCGGAAAACCT3′
β-Actin	F. 5′CACCCGCGAGTACAACCTTC3′
	R. 5′CCCATACCCACCATCACACC3′

### Immunohistochemistry analysis (IHC)

The samples were decalcified in decalcifying liquid, embedded in paraffin and cut into 3 μm sections. The sections were dewaxed in xylene and rehydrated in different concentrations of ethanol. The antigen was repaired by 0.1% trypsin for 30 min at 37 °C. Endogenous peroxidase was blocked with 3% H_2_O_2_. Then sections were blocked with 5% goat serum in PBS for 1 h and then incubated with primary antibody (Ki-67) overnight at 4 °C. After washed three times with PBS, the sections were incubated with secondary antibody for 30 min at 37 °C. Chromogen detection was carried out with a DAB Substrate kit (ZSGB Bio, Beijing, China). The sections were observed and photographed with an inverted microscope (Nikon, Japan).

### Immunofluorescence analysis (IF)

After antigen repaired, the sections were blocked with 5% bovine serum albumin (BSA) in TBS-T for 1 h at room temperature, and then incubated with primary antibodies (LC3, COL1A1) overnight at 4 °C. After washed three times with TBS-T, the sections were incubated with secondary antibody for 1 h at room temperature. The nucleus was counterstained with DAPI stain (Beyotime, Shanghai, China) for 5 min at room temperature. After sealed by anti-fluorescence quencher, the sections were observed and photographed with an inverted microscope (Nikon, Japan).

### Tissue staining

After routine dewaxing and rehydration, the sections were stained with hematoxylin for 5 min, rinsed with tap water, stained with eosin for 2 min and washed again for hematoxylin–eosin (HE) staining to observe the morphological of tissues. Other stainings were progressed based on the instruction manual (Solarbio, Beijing, China). ALP staining was used to detect the number of osteoblast. Goldner trichrome staining was used to examine osteoblastic differentiation. After dehydration of gradient ethanol, and transparention of xylene, the sections were mounted with neutral gum and observed and photographed with an inverted microscope (Nikon, Japan).

### Statistical analysis

All experiments were replicated at least 3 times and achieved similar results. Data were expressed as mean ± SD. All statistical analyses were performed using Social Sciences (SPSS) 22.0 (IBM, Armonk, NY, USA). The independent sample *t*-test was used only for comparison between the two groups, and the measurement of multiple groups was tested by one-way ANOVA. *P* < 0.05 was considered significantly different.

## Results

### GlcN delays bone microstructure destruction and biomechanical properties loss of senile osteoporotic mice

**Fig. 1:**
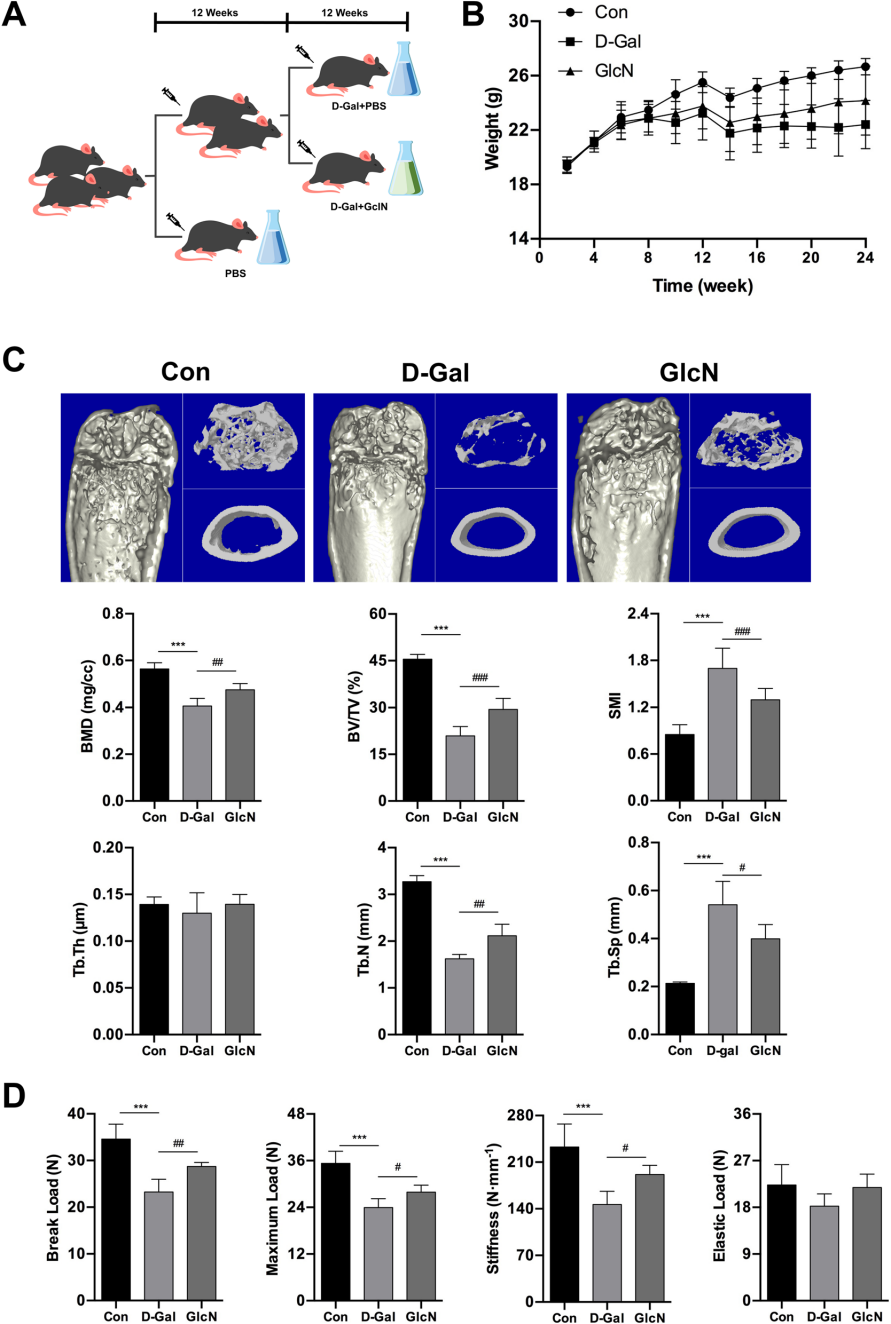
GlcN delays bone microstructure destruction and biomechanical properties loss of senile osteoporotic mice. **A** Schematic representation of the animal grouping. **B** Bodyweight change of mice in each group. **C** The micro-CT images showing representative trabecular bone microarchitecture of the distal femurs and were analyzed to calculate bone micro-architecture parameters, including BMD, BV/TV, SMI, Tb.Th, Tb.N, Tb.Sp for each group. **D** Three-point bending test was used to exhibit the Bone biomechanical properties, including break load, maximum load, stiffness and elastic for each group

In order to explore the effect of GlcN on d-Gal-induced osteoporosis in vivo, we firstly established the senile osteoporosis model in mice by chronic administration of d-Gal (125 mg/kg/day) for 12 weeks, and then additionally treated with GlcN (9 mg/kg/day) for 12 weeks (Fig. 1A). As shown in Fig. 1B, there were no significantly differences between control group and d-Gal group on mice weight after 12 weeks of feeding (*P* > 0.05). However, mice in d-Gal group showed decreased food intake, decreased activity, darkened hair color and hair loss over time. The results of three-dimensional μCT images of femur metaphysis in mice showed that the trabeculae were sparse, irregularly arranged, and widened trabecular spaces in d-Gal group, compared to control group. In addition, the results of µCT data analysis showed that the bone density (BMD, 0.40 ± 0.03 mg/cc), bone volume (BV/TV, 20.94 ± 2.98%) and trabecular number (Tb.N, 1.62 ± 0.09 mm) were significantly decreased, whereas the structural model index (SMI, 1.70 ± 0.26) and trabecular space (Tb.Sp, 0.54 ± 0.10 mm) were significantly increased in d-Gal group, compared to control group (BMD, 0.56 ± 0.03 mg/cc; BV/TV, 45.48 ± 1.53%; TB.N, 3.27 ± 0.13 mm; SMI, 0.85 ± 0.13; Tb.Sp, 0.21 ± 0.01 mm) (***P < 0.001). Meanwhile, the results of µCT data analysis showed that the BMD (0.47 ± 0.03 mg/cc), BV/TV (29.45 ± 3.52%) and Tb.N (2.11 ± 0.24 mm) were significantly increased, whereas the SMI (1.30 ± 0.15) and Tb.Sp (0.40 ± 0.06 mm) were significantly decreased in GlcN group, compared to d-Gal group (^#^*P* < 0.05, ^##^*P* < 0.01, ^###^*P* < 0.001). Interestingly, there were no significantly differences among the three groups on trabecular thickness (Tb.Th, Control, 0.14 ± 0.01 mm; d-Gal, 0.13 ± 0.02 mm; GlcN, 0.14 ± 0.01 mm) (*P* > 0.05) (Fig. 1C). Moreover, the results of three-point bending test showed that the break load (23.25 ± 2.74 N), maximum load (23.93 ± 2.29 N) and stiffness (146.48 ± 19.78 N mm^−1^) were significantly decreased in d-Gal group, compared to control group (break load, 35.32 ± 3.11 N; maximum load, 34.58 ± 3.21 N; stiffness, 232.74 ± 34.61 N mm^−1^) (***P < 0.001). While the maximum load (27.92 ± 1.78 N), break load (28.7 ± 0.89 N) and stiffness (191.33 ± 13.87 N mm^−1^) were significantly increased in GlcN group, compared to d-Gal group (^#^*P* < 0.05, ^##^*P* < 0.01). Interestingly, there were no significantly differences among the three groups on elastic load (control, 22.30 + 3.92 N; d-Gal, 18.22 ± 2.37 N; GlcN, 21.83 ± 2.57 N) (*P* > 0.05) (Fig. 1D). Therefore, we successfully established the senile osteoporosis model in mice by chronic administration of d-Gal. Meanwhile, we also found that GlcN significantly inhibits d-Gal-induced osteoporosis in mice, as shown by GlcN not only significantly improved the microstructure of the distal femur trabecular bone, but also significantly improved the biomechanical properties of the bone.

### GlcN delays senescence of senile osteoporotic mice

**Fig. 2:**
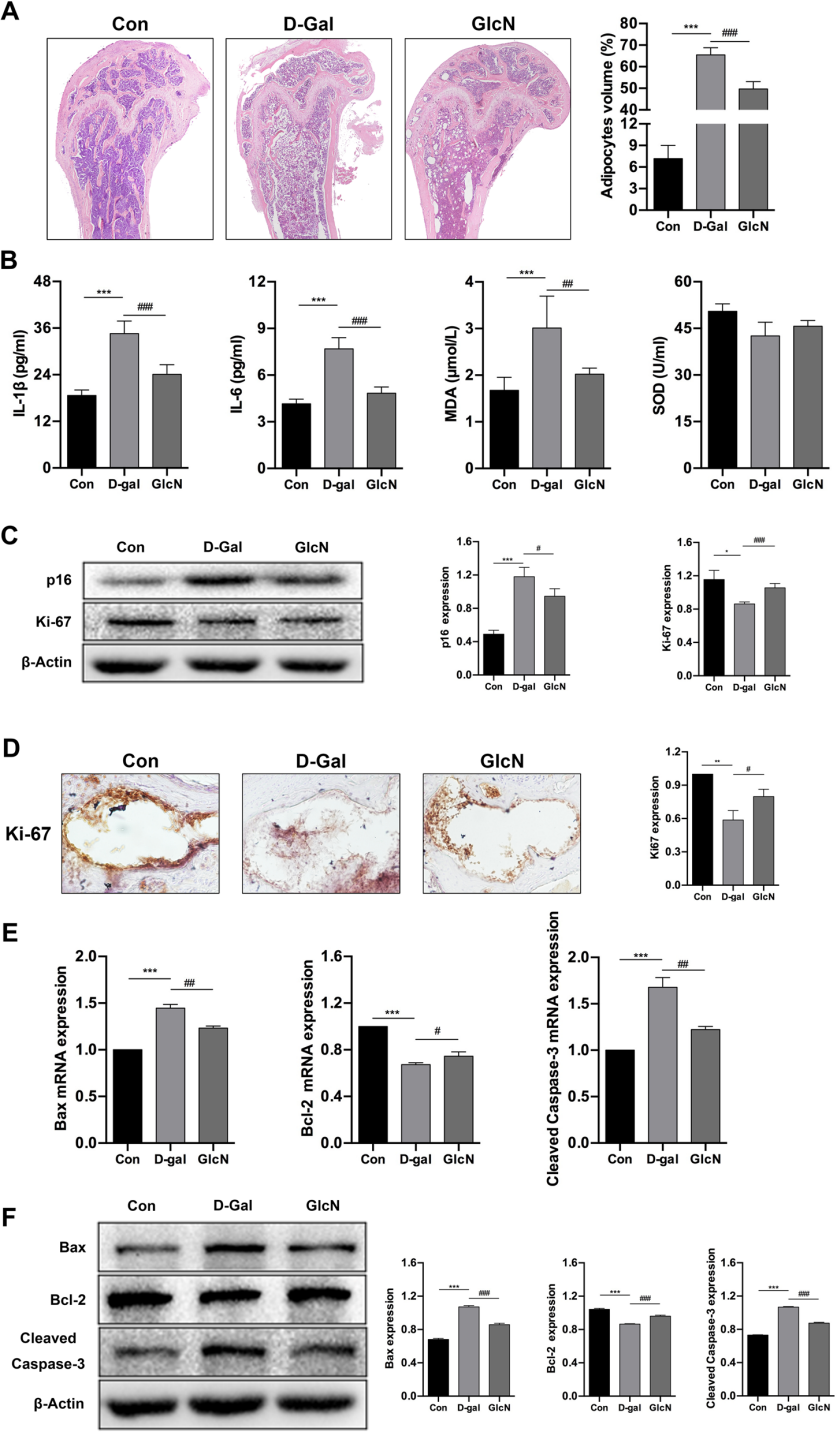
GlcN delays senescence of senile osteoporotic mice. **A** Comparison of adipocytes volume in the femoral bone marrow cavity of each group by HE staining (× 40). **B** Comparison of senescence serum maker expression (IL-1β, IL-6, MDA and SOD) for each group by serum biochemical test. **C** Western blot analysis was used to detect the expression of proliferation marker (Ki-67) and senescence maker (p16). **D** Immunohistochemistry analysis was used to detect the expression of Ki-67 in the distal femur (× 200). **E** Relative mRNAs expression of apoptotic markers (Bax, Bcl-2 and Cleaved Caspase-3) in bone tissue by qRT-PCR. **F** Relative proteins expression of Bax, Bcl-2 and Cleaved Caspase-3 in bone tissue by western blot

In order to further explore the role of GlcN in delaying the progression of d-Gal-induced osteoporosis, we conducted some follow-up studies. The results of HE staining showed that the bone trabeculae in d-Gal group were thin and sparse, and filled with a large number of adipocytes, and presented the phenotype of osteoporosis. We further measured the area of adipocytes per square millimeter of femoral bone trabecular to evaluate the degree of adipogenesis. The results showed that the percentage of adipocytes volume in the femoral bone marrow cavity (65.50 ± 3.27%) was significantly increased in the d-Gal group, compared to control group (7.17 ± 1.84%) (***P < 0.001). Meanwhile, we found that although the percentage of adipocytes volume in the femoral bone marrow cavity (49.67 ± 3.39%) was also increased in GlcN group, but the increased volume was significantly smaller than that in d-Gal group (^###^*P* < 0.001) (Fig. 2A). Next, we detected the senescence-associated indicators in serum. The results showed that the levels of IL-1β (34.58 ± 3.26 pg/ml), IL-6 (7.68 ± 0.73 pg/ml) and MDA (3.01 ± 0.68 μmol/L) (pro-senescence) were significantly increased in d-Gal group, compared to control group (IL-1β, 18.68 ± 1.39 pg/ml; IL-6, 4.15 ± 0.31 pg/ml; MDA, 1.67 ± 0.28 μmol/L) (***P < 0.001). Meanwhile, the levels of IL-1β (24.08 ± 2.49 pg/ml), IL-6 (4.83 ± 0.40 pg/ml) and MDA (2.02 ± 0.13 μmol/L) (pro-senescence) were significantly decreased in GlcN group, compared to d-Gal group (^##^*P* < 0.01, ^###^*P* < 0.001). Interestingly, there were no significantly differences among the three groups on the level of SOD (anti-senescence) (control, 50.48 ± 2.38 U/ml; d-Gal, 0.42.58 ± 4.37 U/ml; GlcN, 0.45.65 ± 1.86 U/ml) (*P* > 0.05) (Fig. 2B). In addition, we detected the expression of senescence-associated proteins in mice bone tissue by WB and IHC. The results of WB showed that the expression of Ki-67 (pro-proliferation/anti-senescence) was significantly decreased, whereas the expression of p16 (pro-senescence) was significantly increased in d-Gal group, compared to control group (*P < 0.05, ***P < 0.001). Meanwhile, the expression of Ki-67 was significantly increased, whereas the expression of p16 was significantly decreased in GlcN group, compared to d-Gal group (^#^*P* < 0.05, ^###^*P* < 0.001) (Fig. 2C). Similar results were found in IHC. The results showed that the expression of Ki-67 was significantly decreased in d-Gal group, compared to control group (**P < 0.01). Although the expression of Ki-67 was also decreased in GlcN group, but the decreased expression of Ki-67 was significantly smaller than that in d-Gal group (^#^*P* < 0.05) (Fig. 2D). Studies showed that apoptosis is closely related to the occurrence and development of senescence-associated diseases, including osteoporosis [24]. Therefore, we detected the expression of apoptosis-associated mRNAs and proteins in mice bone tissue by qRT-PCR and WB, respectively. The results of qRT-PCR showed that the mRNAs of Bax and Cleaved Caspase-3 (pro-apoptosis) were significantly upregulated, whereas the mRNA of Bcl-2 (anti-apoptosis) was significantly downregulated in d-Gal group, compared to control group (***P < 0.001). Meanwhile, the mRNAs of Bax and Cleaved Caspase-3 were significantly downregulated, whereas the mRNA of Bcl-2 was significantly upregulated in GlcN group, compared to d-Gal group (^#^*P* < 0.05, ^##^*P* < 0.01) (Fig. 2E). Similar results were found in WB. The results of WB showed that the proteins of Bax and Cleaved Caspase-3 were significantly upregulated, whereas the protein of Bcl-2 was significantly downregulated in d-Gal group, compared to control group (***P < 0.001). Meanwhile, the proteins of Bax and Cleaved Caspase-3 were significantly downregulated, whereas the protein of Bcl-2 was significantly upregulated in GlcN group, compared to d-Gal group (^###^*P* < 0.001) (Fig. 2F). Taken together, our results demonstrated that GlcN down-regulate the level of aging and apoptosis level in bone tissue of d-Gal-induced osteoporotic mice.

### GlcN delays the progress of osteoporosis in senile osteoporotic mice by promoting autophagy

**Fig. 3:**
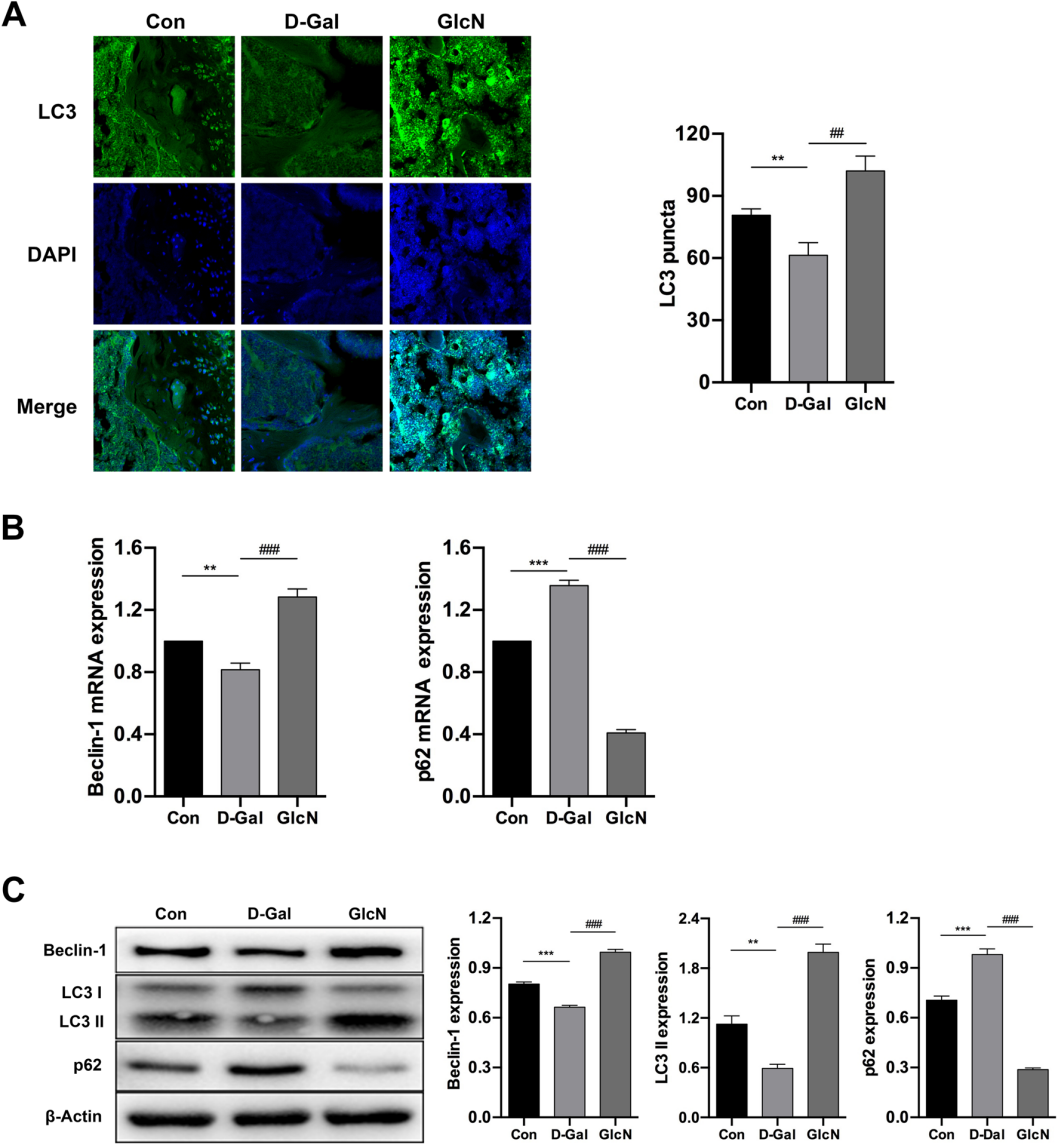
Autophagy participates the anti-osteoporosis effect of GlcN. **A** Representative LC3 immunofluorescence staining (LC3 punctated aggregation and stained with green, DAPI stained with blue) in distal femurs (× 100). **B** Relative mRNAs expression of autophagy-related genes (Beclin-1, p62) in the bone tissue by qRT-PCR. **C** Relative proteins expression of Beclin-1, LC3 and p62 in the bone tissue by western blot

Our previous study demonstrated that GlcN promoted proliferation and inhibited apoptosis of human osteoblasts through upregulating autophagy in vitro [14]. Therefore, our subsequent studies would further explore whether autophagy was involved in anti-osteoporosis of GlcN in vivo*.* We measured IF, qRT-PCR and WB to evaluate the expression of autophagy-related genes and proteins in bone tissue. Studies reported that LC3 is mainly located on the surface of pre-autophagosomes and autophagosomes and is a general biomarker of autophagy [25]. Therefore, we detected the expression of LC3 in proximal tibia by IF. The results of IF showed that the punctate aggregations (autolysosomes) of LC3 was significantly decreased in d-Gal group, compared to control group (**P < 0.001). Meanwhile, the punctate aggregations of LC3 was significantly increased in GlcN group, compared to d-Gal group (^##^*P* < 0.001). Interestingly, we found the punctate aggregations of the LC3 were located on the surface of the bone trabecula (Fig. 3A). As shown in Fig. 3B, the results of qRT-PCR showed that the mRNA expression of Beclin-1 was significantly downregulated, whereas the mRNA expression of p62 was significantly upregulated in d-Gal group, compared to control group (**P < 0.001, ***P < 0.001). Meanwhile, the mRNA expression of Beclin-1 was significantly upregulated, whereas the mRNA expression of p62 was significantly downregulated in GlcN group, compared to d-Gal group (^###^*P* < 0.001). Similar results were found in WB. As shown in Fig. 3C, the results of WB showed that the proteins expression of LC3 II and Beclin-1 were significantly downregulated, whereas the protein expression of p62 was significantly upregulated in d-Gal group, compared to control group (**P < 0.001, ***P < 0.001). Meanwhile, the proteins expression of LC3 II and Beclin-1 were significantly upregulated, whereas the protein expression p62 was significantly downregulated in GlcN group, compared to d-Gal group (^###^*P* < 0.001). Autophagy is a self-protection strategy and plays an important role in the body growth and development by maintaining the body homeostasis, however the level of autophagy is associated with aging. From the above studies, we found that autophagy levels significantly reduced in d-Gal treatment group, however we also found that GlcN up-regulated the level of autophagy to delay the progress of osteoporosis in d-Gal-induced osteoporosis mice.

### Inhibition of autophagy reverses the anti-osteoporosis effect of GlcN

**Fig. 4:**
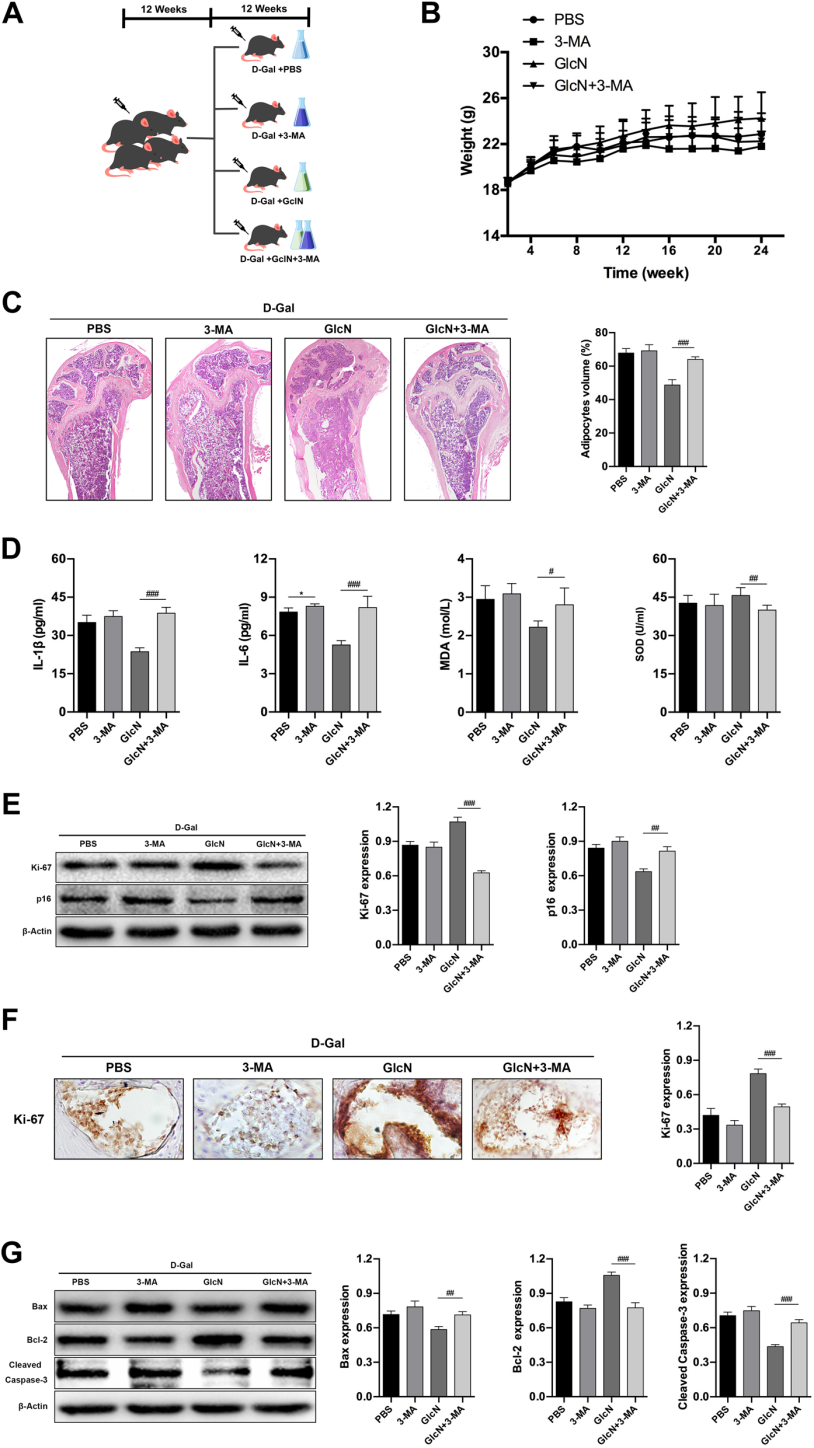
Inhibition of autophagy reverses the anti-osteoporosis effect of GlcN. **A** Schematic representation of the animal grouping. **B** Bodyweight change of mice in each group. **C** Comparison of adipocytes volume in the femoral bone marrow cavity of each group by HE staining (× 40). **D** Comparison of IL-1β, IL-6, MDA and SOD for each group by serum biochemical test. **E** Western blot analysis was used to detect the expression of Ki-67 and p16. **F** Immunohistochemistry analysis was used to detect the expression of Ki-67 in the distal femur (× 200). **G** Relative proteins expression of Bax, Bcl-2 and Cleaved Caspase-3 in bone tissue by western blot

To further clarify the role of autophagy in anti-osteoporosis of GlcN in vivo, the osteoporotic mice were additionally treated with 3-MA (4 mg/kg/day) for 12 weeks (Fig. 4A). There were no significantly differences among the four groups on mice weight after 12 weeks of feeding (*P* > 0.05) (Fig. 4B). Moreover, we evaluated the level of adipogenic differentiation by HE staining. The results showed that the percentage of adipocytes volume in the femoral bone marrow cavity (69.17 ± 3.67%)was significantly increased in the GlcN + 3-MA group, compared to GlcN group (48.67 ± 3.20%) (^###^*P* < 0.001). Meanwhile, there were no significantly differences of the percentage of adipocytes volume in the femoral bone marrow cavity between PBS group (63.92 ± 2.78%) and 3-MA group (64.00 ± 1.55%) (*P* > 0.05) (Fig. 4C). Next, we detected the senescence-associated indicators in serum. The results showed that the levels of IL-1β (43.20 ± 6.84 pg/ml), IL-6 (9.60 ± 1.52 pg/ml) and MDA (3.16 ± 0.22 μmol/L) were significantly increased, whereas the levels of SOD (39.98 ± 1.87 U/ml) was significantly decreased in GlcN + 3-MA group, compared to GlcN group (IL-1β, 23.63 ± 1.55 pg/ml; IL-6, 5.25 ± 0.35 pg/ml; MDA, 2.22 ± 0.16 μmol/L; SOD, 45.65 ± 1.86 U/ml) (^#^*P* < 0.05, ^##^*P* < 0.01, ^###^*P* < 0.001). Meanwhile, there were no significantly differences between PBS group and 3-MA group on the levels of IL-1β (PBS, 19.21 ± 0.23 pg/ml; 3-MA, 37.43 ± 2.27 pg/ml), IL-6 (PBS, 4.35 ± 0.45 pg/ml; 3-MA, 8.32 ± 0.50 pg/ml), MDA (PBS, 2.98 ± 0.75 μmol/L; 3-MA, 3.03 ± 0.38 μmol/L) and SOD (PBS, 42.73 ± 3.97 U/ml; 3-MA, 41.08 ± 5.19 U/ml) (*P* > 0.05) (Fig. 4D). In addition, we detected the expression of senescence-associated proteins in mice bone tissue by WB and IHC. The results of WB showed that the expression of Ki-67 was significantly decreased, whereas the expression of p16 was significantly increased in GlcN + 3-MA group, compared to GlcN group (^##^*P* < 0.01, ^###^*P* < 0.001). Meanwhile, there were no significantly differences of the expression of Ki-67 and p16 between PBS group and 3-MA group (*P* > 0.05) (Fig. 4E). Similar results were found in IHC. The results showed that the expression of Ki-67 was significantly decreased in GlcN + 3-MA group, compared to GlcN group (^###^*P* < 0.001). Meanwhile, there were no significantly differences between PBS group and 3-MA group on Ki-67 expression (*P* > 0.05) (Fig. 4E). Last, we detected the expression of apoptosis-associated proteins in mice bone tissue by WB. The results showed that the expression of Bax and Cleaved Caspase-3 were significantly increased, whereas the expression of Bcl-2 was significantly decreased in GlcN + 3-MA group, compared to GlcN group (^##^*P* < 0.01, ^###^*P* < 0.001). Meanwhile, there were no significantly differences between PBS group and 3-MA group on apoptosis related proteins (*P* > 0.05) (Fig. 4F). In conclusion, our results demonstrated that inhibition of autophagy reverses the anti-osteoporosis effect of GlcN in d-Gal-induced osteoporotic mice.

### GlcN delays osteoporosis by promoting osteoblast autophagy

**Fig. 5.**
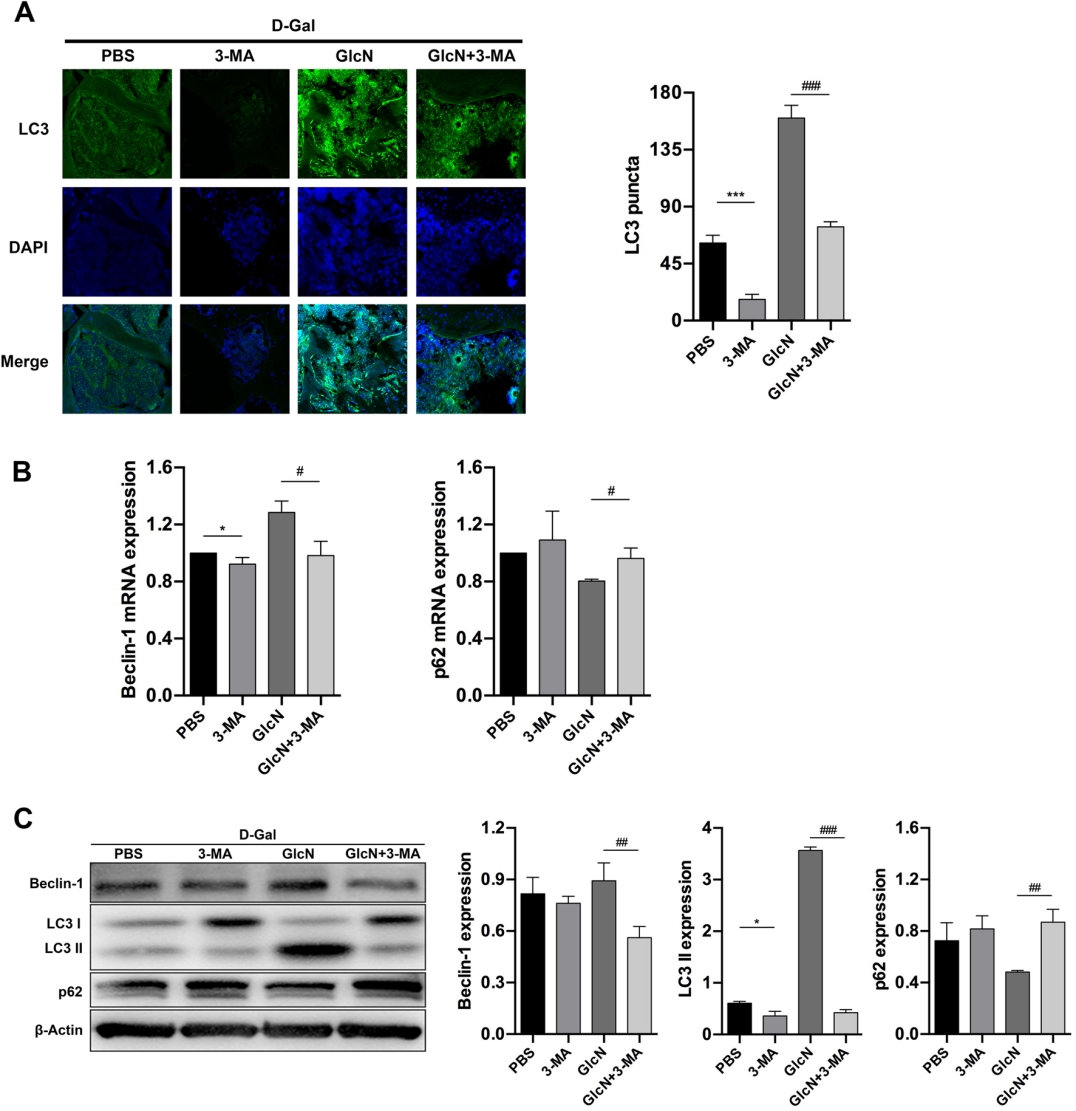
3-MA inhibits the autophagic activation of GlcN. **A** Representative LC3 immunofluorescence staining in distal femurs (× 100). **B** Relative mRNAs expression of Beclin-1 and p62 in the bone tissue by qRT-PCR. **C** Relative proteins expression of Beclin-1, LC3 and p62 in the bone tissue by western blot

**Fig. 6:**
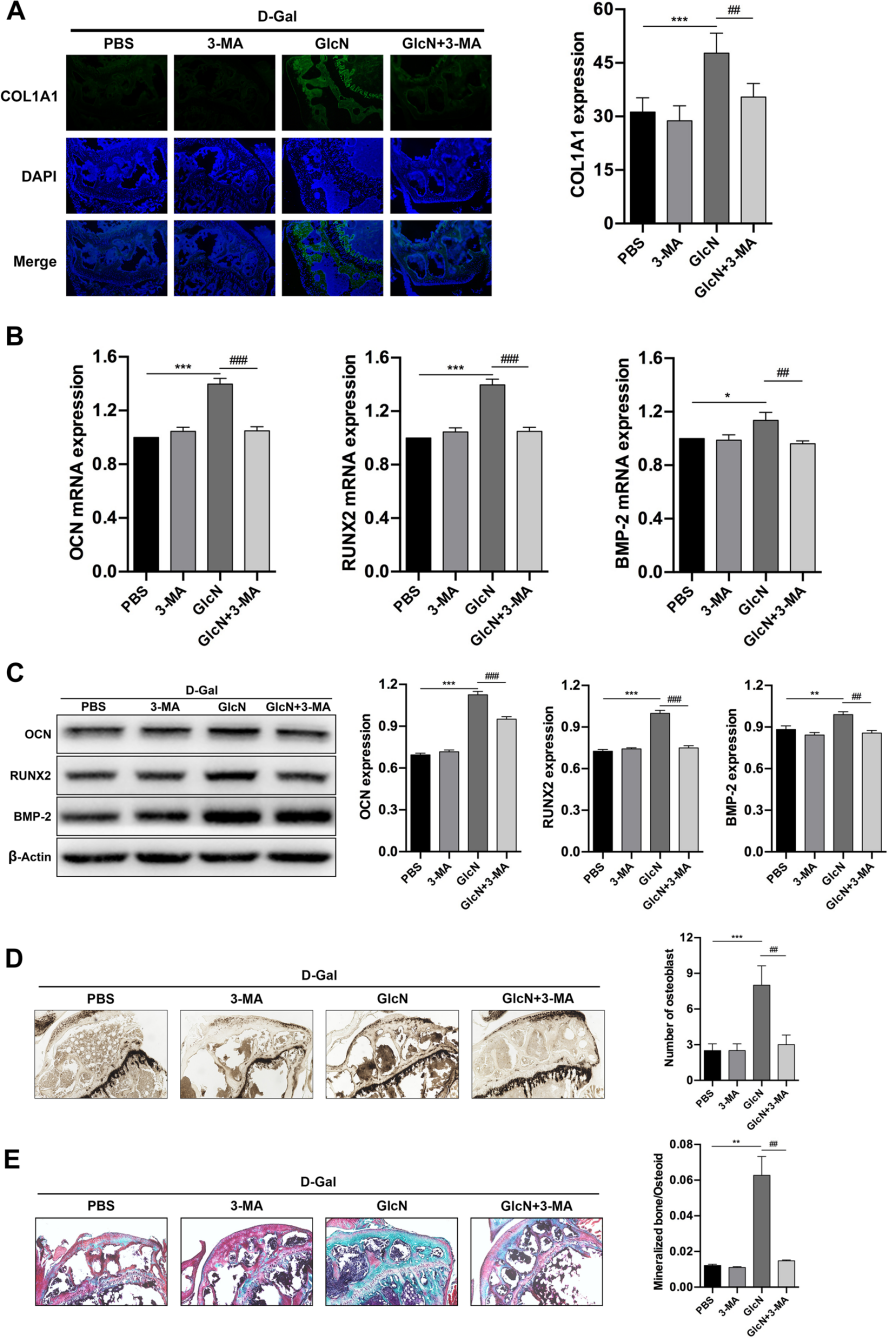
GlcN delays osteoporosis by promoting osteoblast autophagy. **A** Representative COL1A1 immunofluorescence staining (COL1A1 stained with green, DAPI stained with blue) in proximal tibia (× 100). **B** Relative mRNAs expression of osteogenesis-related genes (OCN, RUNX2 and BMP-2) in the bone tissue by qRT-PCR. **C** Relative proteins expression of OCN, RUNX2 and BMP-2 in the bone tissue by western blot. **D** Alkaline phosphatase staining in proximal tibia to exhibit the number of osteoblasts (the cytoplasm of osteoblasts was black granular) (× 100). **E** Goldner trichrome stain in proximal tibia to distinguish osteoid (stained with red) and mineralized bone (stained with green) (× 100)

Along with aging, bone marrow mesenchymal stem cells in bone marrow cavity decreased osteogenic differentiation whereas increased adipogenic differentiation. Our previous study found that GlcN reduced adipogenic differentiation of bone marrow mesenchymal stem cells in senile osteoporotic mice. Meanwhile, we also found that autophagosomes gathered around trabecular bone after GlcN activated autophagy. While osteoblasts were attached around bone trabeculae [26, 27]. In addition, our previous in vitro studies reported that GlcN increased the level of osteoblast autophagy. Therefore, we conducted the follow-up studies to explore whether GlcN exerts an anti-osteoporosis effect by activating osteoblast autophagy. Firstly, we confirmed the inhibition of autophagy did reverse the anti-osteoporosis effect of GlcN by IF, qRT-PCR and WB. IF results showed that the autophagy activation of GlcN was significantly reversed by 3-MA (GlcN group VS. GlcN + 3-MA group, ^##^*P* < 0.01) (Fig. 5A). Furthermore, the results of qRT-PCR showed that the mRNA expression of Beclin-1 was significantly downregulated, whereas the mRNA expression of p62 was significantly upregulated in GlcN + 3-MA group, compared to GlcN group (^#^*P* < 0.05) (Fig. 5B). Similar results were found in WB. As shown in Fig. 5C, the results of WB showed that the proteins expression of LC3 II and Beclin-1 were significantly downregulated, whereas the protein expression of p62 was significantly upregulated in GlcN + 3-MA group, compared to GlcN group (^##^*P* < 0.01, ^###^*P* < 0.001). In addition, the results showed that the protein expression of LC3 II was significantly downregulated in 3-MA group, compared to PBS group (*P < 0.05). Next, we measured IF, qRT-PCR, WB, ALP staining and Goldner staining to evaluate whether GlcN maintains osteogenic activity and promotes differentiation, proliferation and mineralization of osteoblast by activating autophagy, thus exerting anti-osteoporosis effect. The results of IF showed that the expression of COL1A1 (indicator of osteoblast, pro-osteogenesis) was significantly increased in GlcN group, compared to PBS group (***P < 0.001). While the expression of COL1A1 was significantly decreased in GlcN + 3-MA group, compared to GlcN group (^##^*P* < 0.01). Meanwhile, there were no significantly differences between PBS group and 3-MA group on the expression of COL1A1 (*P* > 0.05) (Fig. 6A). The results of qRT-PCR showed that the mRNAs expression of RUNX2, BMP-2 and OCN (indicators of osteogenesis differentiation) were significantly increased in GlcN group, compared to PBS group (*P < 0.05, ***P < 0.001). While the mRNAs expression of RUNX2, BMP-2 and OCN were significantly decreased in GlcN + 3-MA group, compared to GlcN group (^##^*P* < 0.01, ^###^*P* < 0.001). Meanwhile, there were no significantly differences between PBS group and 3-MA group on the mRNAs expression of RUNX2, BMP-2 and OCN (*P* > 0.05) (Fig. 6B). Similar results were found in WB. The results of WB showed that the proteins expression of RUNX2, BMP-2 and OCN were significantly increased in GlcN group, compared to PBS group (**P < 0.01, ***P < 0.001). while the proteins expression of RUNX2, BMP-2 and OCN were significantly decreased in GlcN + 3-MA group, compared to GlcN group (^##^*P* < 0.01, ^###^*P* < 0.001). Meanwhile, there were no significantly differences between PBS group and 3-MA group on the proteins expression of RUNX2, BMP-2 and OCN (*P* > 0.05) (Fig. 6C). As shown in ALP staining (Fig. 6D), the number of osteoblast was significantly increased in GlcN group, compared to PBS group (***P < 0.001). While the number of osteoblast was significantly decreased in GlcN + 3-MA group, compared to GlcN group (^##^*P* < 0.01). Meanwhile, there were no significantly differences between PBS group and 3-MA group on the number of osteoblast (*P* > 0.05). Furthermore, osteoblast mineralization was assessed by Goldner staining. As is shown in Fig. 6E, the osteoblast mineralization was significantly increased in GlcN group, compared to PBS group (**P < 0.01). While the osteoblast mineralization was significantly decreased in GlcN + 3-MA group, compared to GlcN group (^##^*P* < 0.01). Meanwhile, there were no significantly differences between PBS group and 3-MA group on osteoblast mineralization (*P* > 0.05). In summary, the above results demonstrated that GlcN maintains osteogenic activity and promotes osteoblast proliferation and mineralization by activating autophagy, so as to delay the progression of osteoporosis in d-Gal-induced osteoporotic mice.

## Discussion

Although senile osteoporosis is as important as postmenopausal osteoporosis, it is rarely mentioned [28]. Meanwhile, the current research on the pathogenesis of senile osteoporosis mainly focuses on the bone loss caused by the enhancement of osteoclast capacity [29]. However, the occurrence of senile osteoporosis is not only caused by the increase of osteoclast capacity. Decreased osteogenic capacity is also one of the important reasons leading to senile osteoporosis [30]. At present, although there are some drugs that promote osteogenesis in clinics that can be used to treat osteoporosis, the side effects of these drugs severely limit the use time of these drugs [31, 32]. Therefore, it is particularly important to study a drug with clear effects, small side effects, and low price for patients with senile osteoporosis.

Glucosamine (GlcN), a natural monosaccharide, is an important component of the cartilage matrix and has an important role in the prevention of osteoarthritis [33]. GlcN as a dietary supplement has been reported to improve acute kidney injury [34], retinal ischemia–reperfusion injury [35], and osteoarthritis [36] by reducing oxidative damage and inflammatory factor levels. Meanwhile, previous studies showed that GlcN not only effectively protects osteoblasts from oxidative damage caused by hydrogen peroxide, but also prevents bone loss in ovaries removal rat model [11]. However, its mechanism remains unclear. Another study showed that GlcN stimulates the proliferation, activity and differentiation and inhibits osteoblast apoptosis of MC3T3-E1 osteoblast in mice [37]. The results of our previous in vitro study found that GlcN has dual effects on the activity of osteoblasts and is related to autophagy. Low concentration of GlcN not only promoted autophagy and proliferation and reduced apoptosis of osteoblasts, but also maintained osteoblast activity. Moreover, we also found that mTOR signaling pathway was involved [14]. Therefore, we predict that GlcN has a broad application prospect in the treatment of senile osteoporosis, especially in the promotion of osteogenesis. The purpose of this study was to evaluate the therapeutic effect of GlcN on senile osteoporosis in an in vivo animal model.

In this study, d-Galactose (d-Gal) was subcutaneously injected into the back of mice to construct the model of senescent osteoporosis, which was approved by the Animal Ethics Committee of Wenzhou Medical University. After long-term high dose administration, excessive d-Gal converted into galactotol, which caused cellular dysfunction, and cumulative damage leaded to organismal dysfunction and aging [38]. After treatment with d-Gal for 12 weeks, HE staining revealed increased adipose tissue in femoral bone marrow lumen. The results of micro-CT and bone biomechanics showed that bone mass, bone microstructure and biomechanical properties were significantly reduced in the modeling group. This was consistent with previously reported results [39, 40]. To further verify whether d-Gal could accelerate aging, we detected the changes of serum SOD, MDA, IL-1β, IL-6 and other senescence related indexes [41]. The results showed that d-Gal increased the levels of MDA, IL-1β and IL-6. These results suggested that d-Gal accelerated senescence in mice. This was also consistent with previously published research on senescence [39]. Therefore, we successfully established the model of senile osteoporosis by subcutaneous injection of d-Gal in mice.

To investigate the effect and mechanism of GlcN on senile osteoporosis, osteoporosis mice (d-Gal treatment) were respectively given intraperitoneal injection of GlcN (GlcN group) and PBS (d-Gal group). HE staining results showed that GlcN reduced the volume of adipocytes in the bone marrow cavity of distal femur, which suggested GlcN could inhibit the enhancement of adipogenic differentiation of BMSCs induced by senescence. Meanwhile, micro-CT results showed that GlcN inhibited senescence induced bone loss and bone microstructure destruction in the distal femur. In addition, serological detection, western blot and immunohistochemistry results showed significant differences of senescence and apoptosis related indexes between GlcN group and d-Gal group. GlcN could significantly reduce d-Gal-induced senescence and apoptosis. In conclusion, GlcN delayed the progression of senile osteoporosis by delaying bone loss and the destruction of bone microstructure.

Autophagy is an intracellular homeostasis process that degrades and recovers intracellular proteins and damaged organelles through the action of lysosomes to maintain normal cellular functions [42]. A large number of studies have shown that autophagy plays an important role in maintaining bone homeostasis, including regulating cell growth, differentiation and stress response [43]. With age, normal cells suffer from many aging-related diseases due to decreased autophagy [44]. Autophagy increases cell survival by degrading protein, removing damaged intracellular organelle, participating homeostasis and protecting osteoblasts from the accumulation of harmful substances [45]. There are overlapping signal pathways between autophagy and senescence, and the level of autophagy is associated with senescence [46]. Previous studies showed that activation of autophagy decreased adipogenic differentiation and increased osteogenic differentiation of BMSCs, and reversed osteoporotic bone loss [47]. Autophagy levels gradually decrease during senescence. Up-regulation of autophagy slows the senescence process [46] and reduces cell apoptosis[48]. Meanwhile, autophagy plays an important role in osteogenesis [49]. Osteoblasts used autophagosomes as vehicles to secrete apatite crystals and promote bone formation [50]. Ca2^+^ deposition was achieved by activating autophagy to remove misfolded proteins to meet the high energy requirements of the mineralization process [51]. An in vitro study found that the mineralization of osteoblasts could be reduced by knocking out the autophagy-related gene Beclin-1 or by inhibiting the autophagy with autophagy inhibitors [52]. Similar in vivo study also found that Atg7 deficiency impeded osteoblast mineralization and promoted apoptosis in osteoblast-specific Atg7 condition knockout (cKO) mice, while reconstitution of Atg7 could improve ER stress and restore skeletal balance [53]. Therefore, the role of autophagy in promoting osteogenesis can be summarized as follows: (1) promoting BMSCs to the osteogenic differentiation, increasing the number of osteoblasts and maintaining osteogenic activity [54]; (2) reducing the apoptosis of osteoblasts and prolonging the survival time of osteoblasts [55]; (3) participating mineralization process and maintaining bone homeostasis [23]. In summary, autophagy improves osteoporosis symptoms and delays osteoporosis progression of by promoting osteogenesis, which is a feasible way to prevent senile osteoporosis.

To further explore the role of autophagy in GlcN in delaying the progression of senile osteoporosis in mice, autophagy inhibitor 3-MA was used to inhibit autophagy, which blocked the formation of autophagy small bodies and inhibited autophagy activation. From the above results, we found that GlcN increased bone autophagy levels, while 3-MA inhibited autophagy. In addition, 3-MA reversed GlcN’s anti-osteoporosis and anti-apoptosis by inhibiting autophagy. Next, we explored the possibility of GlcN up-regulating osteogenesis by activating autophagy. Current studies have found an increase in COL1A1 expression during osteogenic differentiation [56, 57]. Therefore, COL1A1 can be used as an important indicator of osteogenic ability. Immunofluorescence results showed a decrease in COL1A1 expression in the bones of elderly mice in 3-MA group, compared to GlcN group. In addition, some osteomarkers participated in different stages of osteogenic differentiation. RUNX2 is an early osteogenic differentiation marker, while OPN and BMP2 are late osteogenic differentiation markers [58, 59]. QRT-PCR and Western blot results showed that the mRNAs and proteins expression of RUNX2, OPN and BMP2 were both decreased in 3-MA group, compared to GlcN group. ALP is one of the phenotypic markers of osteoblasts, which directly reflects the activity or function of osteoblasts, and is also the best indicator for evaluating bone mineralization disorders in the body [60]. ALP staining showed that the number of osteoblasts was decreased in 3-MA group, compared to GlcN group. Goldner trichromatic staining also showed the same results. In summary, our results suggested that GlcN may delay the progression of senile osteoporosis by promoting osteogenic autophagy.

The results of the present study suggested that GlcN reduces the bone loss and apoptosis of senile osteoporosis model which caused by d-Gal. Moreover, GlcN promotes osteoblast proliferation and mineralization through enhancing autophagy. Thus, GlcN may be a prospective candidate drug for the treatment of senile osteoporosis, which contributing to the future development of anti-osteoporosis.

## Data Availability

Datasets analyzed during the current study will be made available from the corresponding author upon reasonable request.

## Abbreviations

GlcN: Glucosamine
d-Gal: d-Galactose
3-MA: 3-Methyladenine
BMD: Bone mineral density
IL-6: Interleukin-6
IL-1β: Interleukin-1β
MDA: Malonaldehyde
SOD: Superoxide dismutase
CST: Cell signal technology
HE: Hematoxylin and eosin
ELISA: Enzyme-linked immunosorbent assay
SPF: Specific pathogen-free
Micro-CT: Micro-computed tomography
3-D: Three-dimensional
2-D: Three-dimensional
BV/TV: Bone volume/total volume
Tb.N: Number of trabecular
Tb.Th: Trabecular thickness
Tb.Sp: Trabecular spacing
SMI: Structural model index
PCR: Polymerase chain reaction
DAB: Diaminobenzidine
ALP: Alkaline phosphatase
BMSCs: Bone marrow stromal cells
DAPI: 4′,6-Diamidino-2-phenylindole
TRAP: Tartrate-resistant acid phosphatase
ROS: Reactive oxygen species
